# Reduction of estradiol in human malignant pleural mesothelioma tissues may prevent tumour growth, as implied by in *in-vivo* and *in-vitro* models

**DOI:** 10.18632/oncotarget.9964

**Published:** 2016-06-13

**Authors:** Barbara Nuvoli, Andrea Sacconi, Giancarlo Cortese, Sabrina Germoni, Bruno Murer, Rossella Galati

**Affiliations:** ^1^ Preclinical Models and New Therapeutic Agent Unit, Regina Elena National Cancer Institute, Rome, Italy; ^2^ Oncogenomic and Epigenetic Unit, Regina Elena National Cancer Institute, Rome, Italy; ^3^ SAFU Unit, Regina Elena National Cancer Institute, Rome, Italy; ^4^ Department of Anatomic Pathology, Mestre Hospital, Venezia, Italy

**Keywords:** mesothelioma, estradiol, estrogen receptor α, estrogen receptor β, GPR30

## Abstract

This study aimed to investigate intratumoural estradiol and estrogen-receptors (ERα, ERβ and GPR30) in malignant pleural mesothelioma (MPM) to understand their function. Here, we report that immunohistochemistry of estradiol showed cytoplasmatic staining in 95% of fifty-seven human MPM samples with a trend toward a negative correlation between estradiol levels and the median post-diagnosis survival time. ERβ was only focally positive in 5.3% of cases, GPR30 and ERα were negative in our cases of MPM. GPR30 was detected mainly in glycosylated form in MPM cells. Moreover, G15, a GPR30 antagonist, induced MPM cell death. Altogether, these data suggest that MPM cells produce E_2_ interact with glycosylated forms of GPR30, and this facilitates tumour growth. Estradiol was found in MPM cells and plasma from mice mesothelioma xenografts. Concurrent reduction in tumour mass and plasmatic estradiol levels were observed in the mice treated with exemestane, suggesting that the reduction of E_2_ levels inhibit MPM growth. Thus, it appears that agents reducing estradiol levels could be useful to MPM therapy.

## INTRODUCTION

Mesothelioma is a rare type of cancer that arises from mesothelial cells within the pleura, the peritoneum, the pericardium, and the tunica vaginalis. The main cause is exposure to asbestos. The pleura is the most frequently diseased organ seen in about 80% of cases. Malignant pleural mesothelioma (MPM) is an aggressive tumour that is resistant to conventional treatment including chemotherapy, surgery or radiation [1]. The median survival of patients is generally short once mesothelioma is diagnosed; untreated and treated patients survive between 4 to 13 and 6 to 18 months, respectively [1, 2]. Research on the molecular pathways involved in the development of mesothelioma should yield important information that could drive therapeutic strategies in the near future [1].

Aromatase (CYP19A1), the enzyme involved in the synthesis of estrogen, was biologically active in MPM cell lines. Furthermore, in a large group of human MPM samples it results that CYP19A1 was expressed as a cytoplasmic protein and its expression was significantly associated with poor survival of patients [3]. The role of estrogens in different human tumours is still controversial and evolving [4]. In lung, estrogens contribute to differentiation and maturation and in lung tumours also stimulate growth and progression of lung tumours [5–7]. Estrogen mediates a plethora of biological processes via intracellular receptors located in the cytoplasm or on the nuclear membrane through two main pathways referred to as “genomic” and “non-genomic” [8]. The genomic mechanism involves the binding of estrogen-receptor complex to hormone-responsive elements, specific sequences of the DNA, resulting in the transcriptional regulation of the genes encoding associated proteins. The non-genomic mechanism allows the cell to use proteins already present. This action occurs rapidly but the effect can be long lasting. Processes, such as proliferation, survival, apoptosis, and other functions in diverse cell-types are regulated by non-genomic mechanisms [8]. Steroid hormone modulation of ion channels and G-protein-coupled receptors (GPCR) has been shown in diverse tissues [9]. G protein-coupled receptor 30 (GPR30) also known as G protein-coupled estrogen receptor 1 (GPER), binds estrogen and cause estrogen-mediated adenylyl cyclase stimulation (which produces Cyclic adenosine 3′,5′-monophosphate (cAMP)) and phosphoinositide 3-kinases (PI3K) cascade. Exemestane, a steroidal inhibitor of CYP19A1, was found to be effective in inhibiting proliferation of MPM cell lines and in the treatment of nude mice carrying MPM [3, 11]. This did suggest that estrogen and estrogen receptors (ERs) could be involved in the treatment of MPM supporting further investigation on exemestane and new compounds, which are active with the same mechanism of action [10].

Interestingly, exemestane induces cell death in MSTO-211H (MSTO) by PI3K and cAMP inhibition, pathways involved in the action of GPR30. Intuitively, it can be hypothesized that the action of exemestane is achieved by reducing the levels of E2, its binding with GPR30 and related downstream pathway [11]. In MPM, the discussion on ERα and ERβ expression is controversial, while nothing is known of GPR30 [12, 13]. In the present study, we aimed at examining the role of genomic and especially non–genomic action in MPM and its clinical and biological significance, estradiol (E2), ERα, ERβ and GPR30 were investigated by immunohistochemistry in 57 human MPM samples. In addition, E2 was evaluated in normal (Met5A) and five malignant mesothelium (MSTO, NCIH-2452 (NCI) Ist-Mes1, Ist-Mes2 and MPP89) cell lines, and plasma from mice mesothelioma xenografts treated with exemestemane. GPR30 expression was studied on normal and malignant mesothelium cell lines by western blot (WB) and its activity was tested in MPM cell lines using the GPR30 receptor antagonist, namely G15.

## RESULTS

### Normal pleura and malignant pleural mesothelioma tissues endowed E_2_

**Figure 1:**
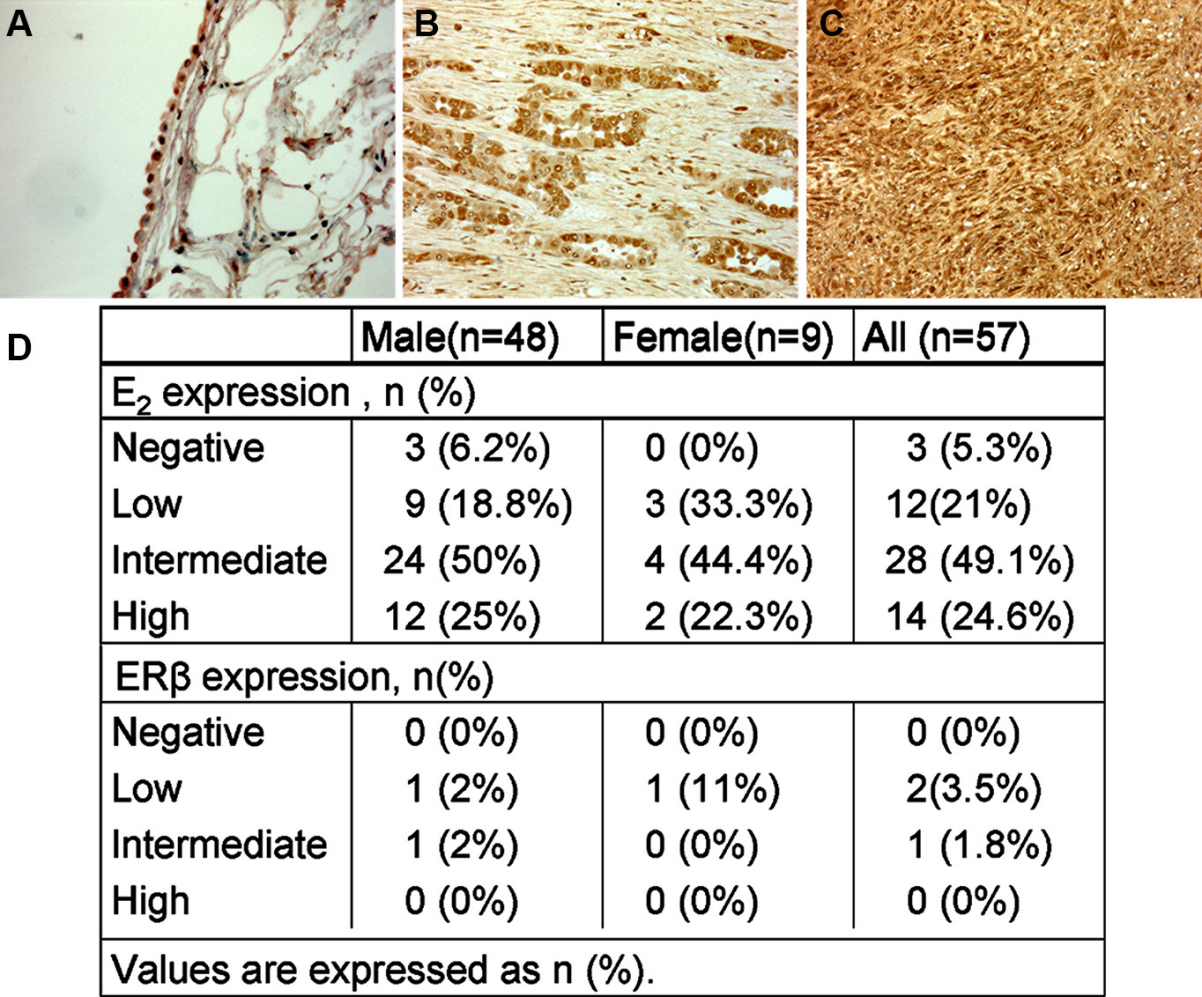
E_2_ expression by immunohistochemistry in normal pleural and MPM (**A**) Normal mesothelium cells showing a cytoplasmatic staining for E_2_, Original magnification, × 100 (**B**) and (**C**) the neoplastic cells express a strong cytoplasmatic positive reaction for E_2_ in both epithelioid and sarcomatous mesothelioma, Original magnification, × 200. (**D**) Percentage of E_2_ and ERβ staining in human tumour samples.

E_2,_ ER, ERα, ERβ and GPR30 expression were determined using immunohistochemistry in tumour biopsy specimens for a well-defined cohort of MPM subjects with more than a 5 year post-diagnosis follow-up. Paraffin-embedded tumour tissue samples from 57 patients ranging between the age of 45 to 80 (clinicopathologic characteristics are reported in Table 1) and 5 normal control subjects were analyzed. We found cytoplasmatic staining for E_2_ in all normal pleura and in 54 mesothelioma samples (Figure 1A, 1B, 1C) with different proportion of positively stained cells between the tumour specimens (Figure 1D). High staining was observed in 14 of 57 (24.6%) tumour samples, intermediate in 28 out of 57 (49.1%), low in 12 out of 57 (21%) and negative in 3 out of 57 (5.3%) tumour tissues (Figure 1D).

**Table 1:** Characteristics of the patients enrolled in the study

Patients	Histotype	Therapy	Survival	Status Survival	Age	Sex
1	Epithelioid	TP + CT + RT	42	D	59	M
2	Epithelioid	TP + CT	8	D	67	M
3	Sarcomatoid	No	3	D	80	M
4	Epithelioid	CT + RT	13	D	76	M
5	Epithelioid	TP + CT + RT	11	D	70	M
6	Epithelioid	TP + CT + RT	38	D	63	M
7	Epithelioid	PPE + CT	13	D	65	M
8	Epithelioid	PPE + CT	9	D	67	M
9	Epithelioid	TP + CT	17	D	71	M
10	Epithelioid	PPE + CT + RT	11	D	55	M
11	Epithelioid	CT + RT	8	D	75	F
12	Biphasic	TP + CT + RT	25	D	71	M
13	Biphasic	TP + CT + RT	18	D	69	M
14	Epithelioid	PPE + CT	9	D	68	M
15	Epithelioid	PPE + CT	10	D	68	M
16	Epithelioid	TP + CT + RT	26	A	77	F
17	Sarcomatoid	No	2	D	75	M
18	Epithelioid	TP + CT + RT	24	D	62	M
19	Epithelioid	TP + CT + RT	18	D	62	F
20	Epithelioid	TP + CT + RT	23	D	45	M
21	Epithelioid	TP + CT + RT	25	D	46	M
22	Biphasic	TP + CT + RT	18	D	77	F
23	Sarcomatoid	CT + RT	15	D	72	M
24	Epithelioid	TP + CT	14	D	76	M
25	Epithelioid	TP + CT + RT	16	D	69	M
26	Epithelioid	TP + CT	16	A	66	M
27	Epithelioid	TP + CT + RT	11	D	68	M
28	Epithelioid	TP + CT + RT	17	D	64	M
29	Sarcomatoid	CT	6	D	73	M
30	Epithelioid	TP + CT	14	D	69	F
31	Epithelioid	TP + CT + RT	9	D	76	M
32	Epithelioid	TP + CT + RT	6	D	79	M
33	Epithelioid	TP	5	D	77	M
34	Epithelioid	TP + CT + RT	27	D	65	M
35	Epithelioid	TP + CT + RT	22	D	59	M
36	Epithelioid	TP + CT + RT	14	D	58	M
37	Epithelioid	TP + CT	8	D	76	M
38	Sarcomatoid	No	5	D	77	M
39	Epithelioid	PPE + CT	10	D	66	M
40	Epithelioid	TP + CT + RT	15	D	64	M
41	Biphasic	TP + CT	8	D	63	M
42	Epithelioid	PPE + CT	13	D	68	M
43	Epithelioid	TP + CT	19	D	62	M
44	Epithelioid	TP + CT	27	D	66	F
45	Epithelioid	TP + CT + RT	11	D	72	M
46	Epithelioid	CT + RT	7	D	75	M
47	Epithelioid	TP + CT + RT	15	D	70	M
48	Biphasic	TP + CT + RT	13	D	69	M
49	Epithelioid	PPE + CT	9	D	65	M
50	Epithelioid	PPE + CT	10	D	68	M
51	Epithelioid	TP + CT + RT	16	D	67	F
52	Sarcomatoid	No	5	D	74	M
53	Epithelioid	TP + CT + RT	20	D	62	M
54	Epithelioid	TP + CT + RT	19	D	62	F
55	Epithelioid	TP + CT + RT	23	D	54	M
56	Epithelioid	TP + CT + RT	25	D	64	M
57	Biphasic	TP + CT + RT	18	D	67	F

TP = Total Pleurectomy, CT = Chemotherapy, RT = Radiotherapy, PPE = Pleuropnemonectomy, A = Alive, D = Dead.

ERβ was slightly positive in normal pleura and only focally positive in 3 cases of MPM, 2 epithelioid and 1 biphasic histotype. The epithelioids were positive while the sarcomatoid was negative for E_2_ at the time of diagnosis. Both hystotypes received therapy and the survival time was 17 and 27 months for epithelioid (E_2_+) and 25 months for the biphasic (E_2_−). For the small number of positive samples for ERβ no correlation with other variables can be relied upon. GPR30 has a cytoplasmatic expression in normal mesothelium, but not in our cases of MPM. Alpha ERα was negative in both normal and malignant mesothelial cells.

### E_2_ level inversely correlates with the median post-diagnosis survival time

**Figure 2:**
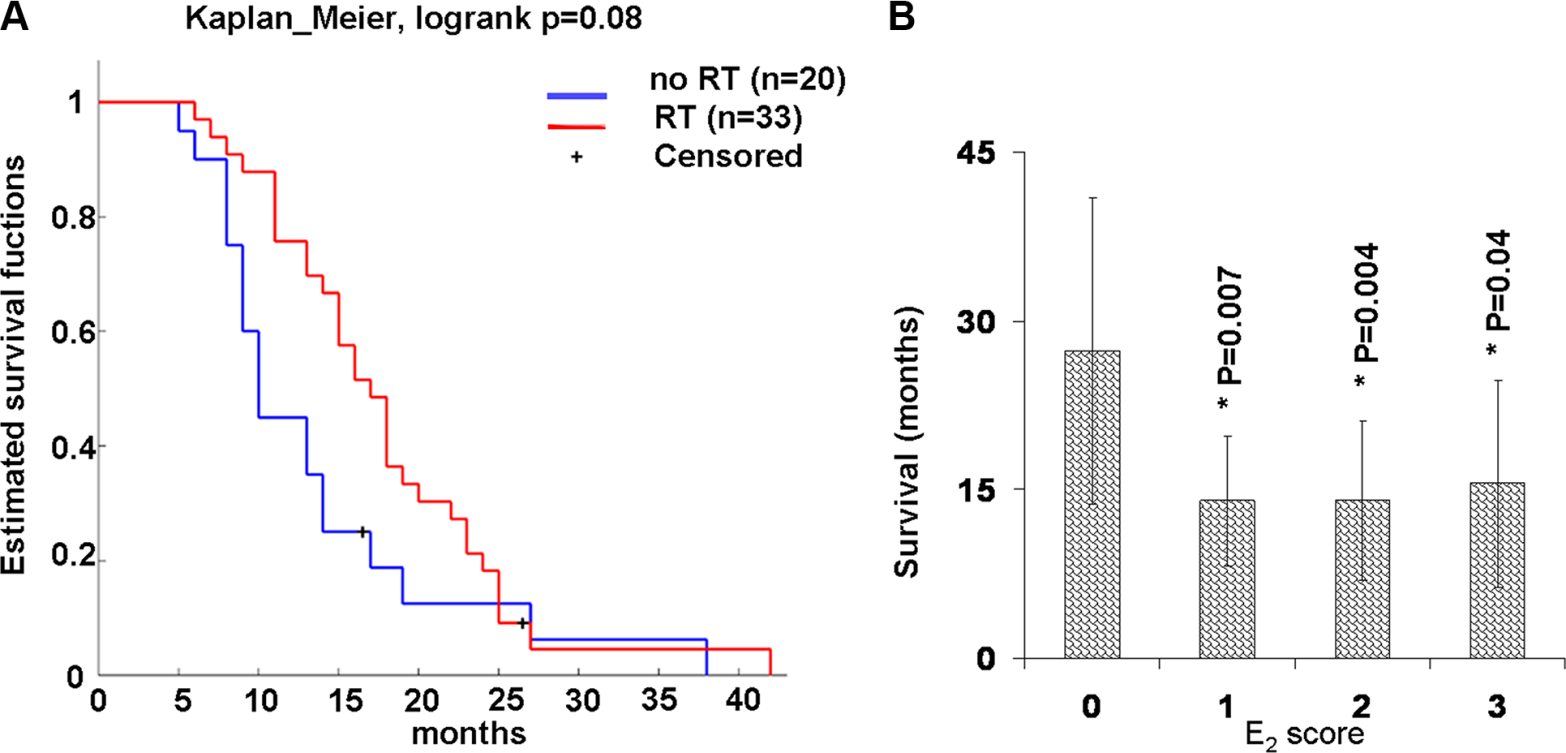
Survival plot by therapy and E_2_ expression in human samples (**A**) Kaplan-Meier survival plot by therapy. All subjects are dead except two (censored) still alive at the time of the study. They received either chemotherapy and radiotherapy (RT) or chemotherapy alone (without RT) post-diagnosis. (**B**) E_2_ expression and median postdiagnosis survival time. Graph shows the median post-diagnosis survival time of all human samples tested by immunohistochemistry for E_2_ and E_2_ score. E_2_ score = 0 (negative), 1 (up to 30%), 2 up to 60% and 3 more that 60% of positive cells. Values were reported as median ± standard deviation, * indicate significant differences (*p* < 0.05) versus E_2_ score = 0 calculated by Student's test.

Table 1 summarizes the distribution of gender and established clinicopathological factors. The gender distribution of the MPM cohort was 48 male and 9 female subjects; the median age at diagnosis was 67 years for male subjects and 69 years for female subjects. Histological types were determined to be 45 (79%) epithelioid, 6 (10.5%) biphasic, and 6 (10.5%) sarcomatoid. The majority of the subjects (52) had received cisplatin-based chemotherapy post-diagnosis, 34 of these (65%) also received radiotherapy. Differences in the therapy were not statistically significant on survival (Figure 2A).

We observed median survival times for patients with low, medium and high E_2_ levels lower than patients without E_2_ (Figure 2B). Although it is an unusual representation, we preferred to use a histogram. This allows us to highlight a significant difference (*P* < 0.05) between patients with low, intermediate and high E_2_compared to patients without E_2_ that the Kaplan-Meier survival plot would neglect. In fact, although this is a simple observation it is important to provide communication supported by the strong results obtained *in vivo* experimental model described below.

Extrapolating the probability of survival after 2 years of follow-up from Table 1 was 67% for subjects without E_2_ and 13% for subjects with low, intermediate and high E_2_ levels. The probability of survival after 2 years of follow-up was 11% for females (1♀/9♀) and 17% (8♂/48♂) for males, significant differences between the gender compared to its median survival times were not observed.

### E_2_ levels in *in vitro* and *in vivo* MPM experimental models

**Figure 3:**
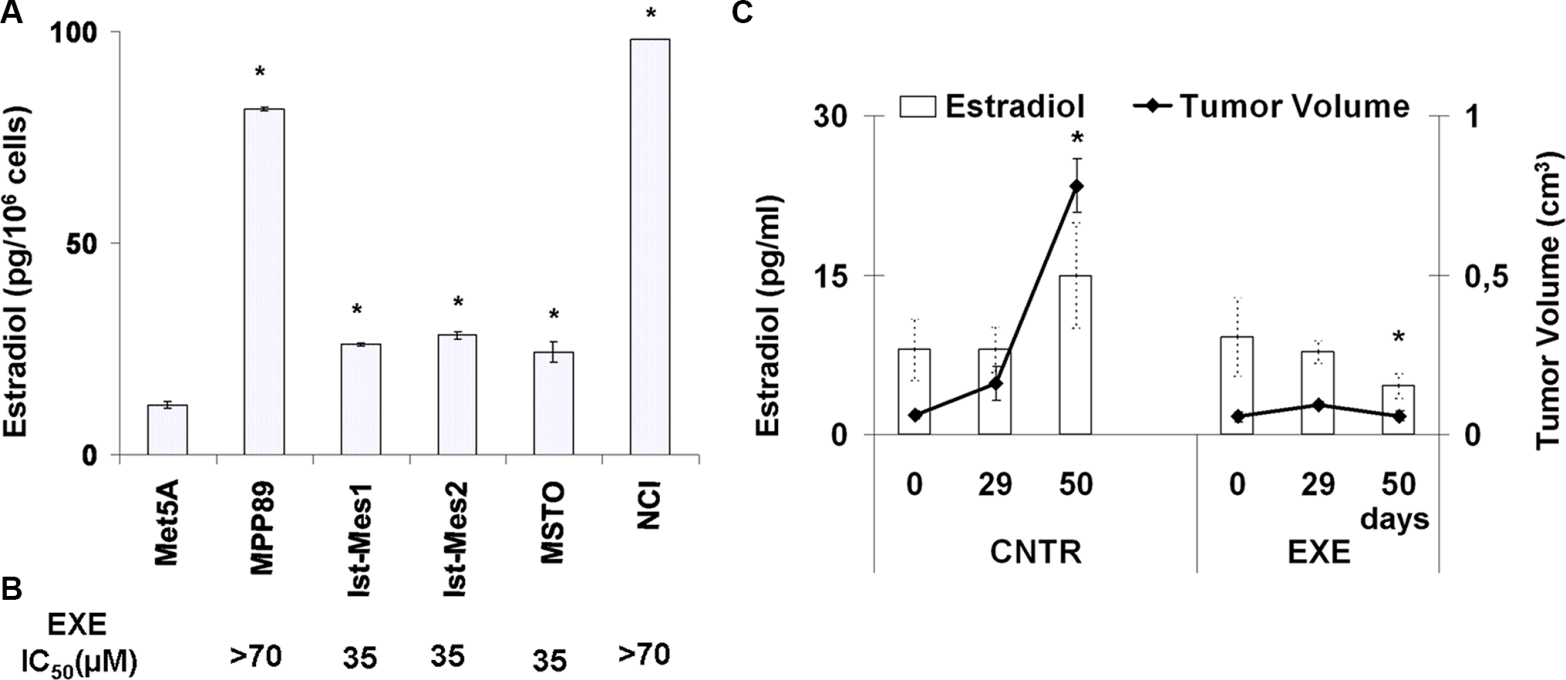
Evaluation of E_2_ levels on normal and malignant mesothelium (**A**) Graph shows the levels of E_2_ in normal mesothelio (Met5A) and five MPM (MPP89, Ist-Mes1, Ist-Mes2, MSTO and NCI) cell lines in basal condition. Values were reported as median ± standard deviation of three different experiments, *indicate significant differences (*p* < 0.05) vs Met5A. (**B**) In correspondence of each single MPM cell lines is indicated exemestane (EXE) IC_50_, expressed as μM. (**C**) Plasmatic E_2_ levels in mice mesothelioma xenograft. Graph summarizing the plasmatic E_2_ levels (left axis) and tumour volume (right axis) at three different time in two groups of mice carrying MPM: control (CNTR) and treated (EXE). Time 0 was before of the start of exemestane treatment. CNTR was untreated and EXE was treated with exemestane as described in materials and methods. Each value represents the average and standard deviation. *indicate significant E_2_ levels and tumour volume differences (*p* < 0.05) vs CNTR at 50 days.

E_2_ levels were quantified in normal and malignant mesothelium cells (Figure 3A). The normal Met5A showed E_2_ levels significantly less than the MPM cell lines (*P* < 0.05). It was not possible to detect E_2_ after treatment of MPM cell with exemestane, probably due to the sensitivity of methods adopted (5 pg/ml). Interesting, Ist-Mes1, Ist-Mes2 and MSTO lines that were more sensitive to exemestane exhibited lower levels of E_2_. In Figure 3B, the IC_50_ (concentration of a drug required for 50% inhibition *in vitro*), of MPM cell lines was reported.

In order to evaluate the role of E_2_ on tumour growth *in vivo*, we used a MPM xenograft animal model resulting from the subcutaneous injection of MSTO cells. After the establishment of palpable lesions mice were assigned to two groups of treatment: control and exemestane as described above.

We determined the plasmatic E_2_ levels and tumour growth at different times 0, 29 and 50 days (Figure 3C). Plasmatic E_2_ levels and tumour volume in the CNTR group at 50 days increased significantly, *P* = 0.001192 and 1.5 × 10^−6^, respectively versus the time 0. Vice versa plasmatic E_2_ levels in the EXE group at 50 days decreased significantly (*P* = 0.0183) versus the time 0. Due to the effectiveness of the therapy, no significant value for tumour volume was calculated at 50 days versus 0 day. By comparing the CNTR and EXE groups at the time 50, a significant difference in the E_2_ levels (*P* = 0.000509859) and tumour mass (*P* = 9.99382E-07) was highlighted, suggesting that there was a positive correlation between plasmatic E_2_ levels and tumour volume.

### GPR30 and E_2_ are involved in mesothelioma cell proliferation

**Figure 4:**
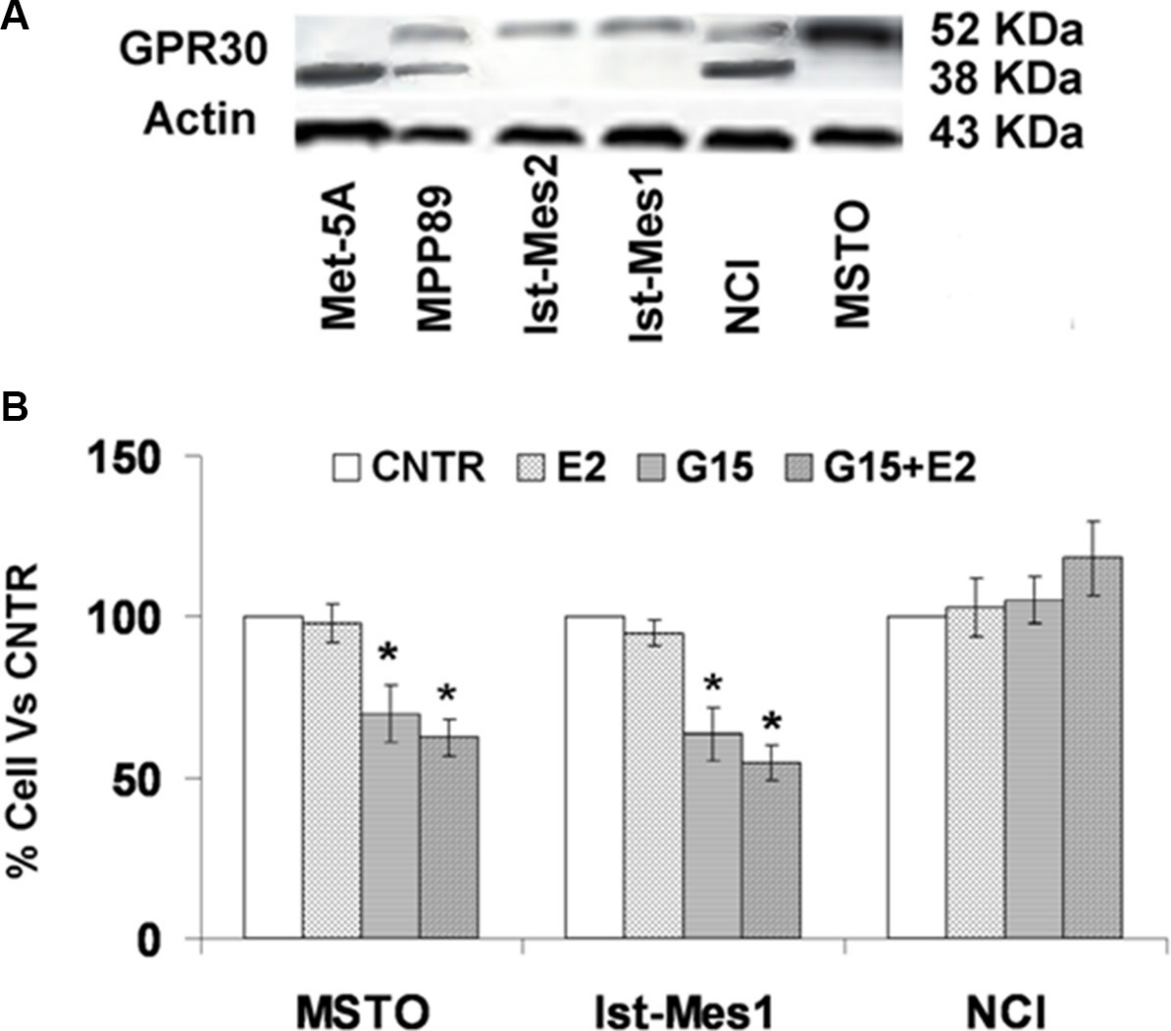
GPR30 protein expression in normal and malignant mesothelium cells and cell viability on MPM cells treated with G15 or E_2_ with G15 (**A**) GPR30 protein expression was found in Met5A as non glycosylated form at approximately 42 kDa, in Ist-Mes2, Ist-Mes1, and MSTO as glycosylated form at about 52 kDa and in NCI and MPP89 as glycosylated and non glycosylated form. Actin was used as loading control. (**B**) Graph summarizing the percentage of cell viability resulting upon exposure for 48 h of MSTO, Ist-Mes1 and NCI cells to E_2_ (10 nM) or G15 (10 nM) or E_2_ (10 nM) with G15 (10 nM), versus CNTR (untreated). Values were reported as median ± standard deviation of three different experiments, *indicate significant differences (*p* < 0.05) vs CNTR.

GPR30 protein expression was found in normal and malignant mesothelium cells (Figure 4A). The molecular weight (MW) of GPR30 is estimated to be 42 kDa, but higher MW sizes have been reported due to glycosylation and interaction with other proteins [14]. GPR30 protein expression was predominantly in a non-glycosylated form in Met5A, as glycosylated form in Ist-Mes2, Ist-Mes1 and MSTO, glycosylated and non-glycosylated form in MPP89 and NCI. Interestingly, cell lines with increased sensitivity to exemestane (Figure 3B) were those consisting of the glycosylated GPR30 form only. Using RNAi silencing and G15, a selective GPR30 antagonist, it is possible to demonstrate the involvement of GPR30 in cell proliferation. The methods are comparable and lead to the same results and therefore we used G15 for our experiments [15]. In order to test the role of GPR30 in MPM proliferation, we chose three MPM cell lines, MSTO and Ist-Mes1 with glycosylated form and NCI with glycosylated and non-glycosylated form of GPR30. Initially, we calculated the concentration of E_2_ (10 nM) which does not cause cell death and then we tested the effect of G15 alone and in association with E_2_ (Figure 4B). G15, alone and in association with E_2,_ induced death cellular in MSTO and Ist-Mes1 while no effect was evident in NCI. Being G15 an GPR30 antagonist it was predicted that GPR30 and E_2_ were required for proliferation in Ist-Mes1 and MSTO.

## DISCUSSION

To the best of our knowledge, this is the first report to have shown intratumoural concentrations of E_2_ in MPM. Estrogen controls primarily the growth, maturation and function of the female reproductive apparatus. Estrogen also influences hormone-related cancers such as breast, endometrial, prostate, ovarian and thyroid cancers [16]. Estrogenic actions have been postulated to contribute to the development and/or progression of non-small cell lung carcinoma (NSCLC) [17, 18]. The intratumoural concentration of E_2_, the most biologically active estrogen, was significantly higher in NSCLC than non-neoplastic lung tissues [19]. Pinton et al. found the expression of ERβ (but not ERα) in patients with malignant pleural mesothelioma. They support ERβ action as tumours suppressor and independent predictor of improved survival [12]. Two other reports deal with ERs in malignant mesothelioma. One recognizes malignant mesothelioma as ER negative and another reports a statistically significant enhancement of estrogen receptor 1 (ERS1) methylation in malignant mesothelioma compared to non-tumour lung samples [19, 20]. The role in normal pleura and MPM of the ERs is not so clear, and also data on the expression of E_2_ and GPR30, are not available. In the present study, E_2_, ERα, ERβ and GPR30 were investigated by immunohistochemistry on 57 human MPM samples within the age range of 45 to 80 year and 5 normal control subjects. Slight positive ERβ staining in normal pleural specimens and focally positive in only 3 cases of MPM were observed, 2 males of 48 (4%) and 1 female of 9 (11%). Compared to a previous report where staining of ERβ was detected in 8 males of 78 (13.6%) and 4 females of 19 (21.1%) our investigation brings a lower number of positivity [12]. Generally, discrepancies between immunohistochemical studies may relate to different populations studied, tissue handling and processing, specificity and sensitivity of the primary antibody, characteristics of the detection system, criteria used in the evaluation process, etc. In agreement with the data in literature, no staining for ERα was found in MPM tissue samples [12, 13]. Cytoplasmatic GPR30 staining was observed in normal mesothelium, but not in our mesothelioma cases. This result was unexpected because, in a previous study, we supposed that GPR30 can be involved in exemestane response since it acted by cAMP modulation and estrogens and antiestrogens signal via GPR30 was mediated by stimulation of cAMP [11, 21] To verify this hypothesis, we explored the GPR30 expression in normal and mesothelioma cell lines by western blot and conducted *in vitro* experiments to test the GP30 function with G15, its antagonist. We detected non-glycosylated forms of GPR30 in normal mesothelium cells (Met-5A), glycosylated form in MPM cells that are more sensitive to exemestane (Ist-Mes1, Ist-Mes2 and MSTO) and glycosylated and non-glycosylated form in MPM cells that are less sensitive to exemestane (MPP89 and NCI). MPM cells upon G15 treatment responded differently. MSTO and Ist-Mes1, expressing glycosylated GPR30, were killed by G15 and the addition of E_2_ with G15 did not change the result. In NCI, where the prevalent form of GPR30 was non-glycosylated, the treatment with G15 as well the association E_2_ with G15 did not induce cellular death. This means that different forms may have different activities, In cell lines expressing GPR30 glycosylated form E_2_ is required to proliferate. During protein synthesis, glycosylation guides the correct folding of the receptor, therefore it is possible that the specificity/affinity of GPR30 antibody may vary between different methods [22, 23]. Again, the characteristics of biomolecules can change during the time between taking the sample from the patient when the sample is processed. The human samples tested in this study were stored from previous years thus we do not know whether the correct pre-analytical procedures were ever applied. To verify this assumption, the collection of fresh MPM tissue was undertaken. In immunohistochemistry only the unglycosylated forms are visible, while in western blot where the proteins were extracted from fresh cell culture, glycosylated and non-glycosylated forms are detectable. In light of what the results may show they might not be contradictory because both methods show the non-glycosylated form in normal samples. Further studies are needed to investigate this assumption. However, on the other hand, it is the first study of GPR30 expression in mesothelioma. Interestingly, in literature the debate on GPR30 remains open. In breast cancer tissue, GPR30 downregulation was associated with poor clinical outcome [24] and GPR30 expression decreased from benign to malignant ovarian tumours [25]. Other studies also suggest that increased GPER correlates with disease severity and reduced survival in ovarian, breast and endometrial cancers [26–28]. However, there is disagreement regarding the pharmacological profile and the subcellular localization of GPR30, which indicates that still some details of the receptors are not known [29].

To complete the study, we investigated the levels of E_2_ in normal and malignant mesothelium tissue and cell lines and in mice mesothelioma xenografts. We found cytoplasmatic staining for E_2_ in all normal pleura and in 54 mesothelioma samples with different proportion of positively stained cells between the tumour specimens. Negative staining was observed in 5.3%, low in 21%, intermediate in 49.1% and high in 24.6% of cases. No correlation was possible between E_2_ levels and ERβ for the small number of ERβ. Median survival times for patients enrolled in the study was of 14 months. When the cohort of patients was divided according to the E_2_ levels, we noted that median survival times for patients low, intermediate and high E_2_ levels were significantly less than the patients without E_2_ levels (*P* < 0.05), 27 months for negative E_2_ levels and about 14 for others (Figure 2A). Therefore, we found a trend toward a negative correlation between E_2_ levels and the median post-diagnosis survival time. We use the term “trend”, because even the subtlest statistical analysis is not feasible because the samples with negative levels of E_2_ are very few. On the other hand, with these percentages of E_2_ negative, also extending the analysis to other new materials, it is not certain that the number of samples is sufficient for statistical analysis. Also the difficulties in finding new and large number of mesothelioma samples are not to be neglected since this type of cancer is not very common. Nevertheless, we hope that it paves the way for further research on the hormonal role in the treatment of MPM. This study covers patients that have had oncological treatment after surgery including total pleuroctomy and pleuropnemonectomy. Several studies with trimodality therapy (surgery, radiation, and chemotherapy) have reported results with rather similar outcomes: overall median survival ranges from 14 to 28 months [30, 31]. The overall median survival of our patients was 15 months. Significant differences on survival time was not found between MPM patients undergoing radiotherapy alone (17.5 months) or in combination with chemotherapy (10 months) after surgery. Moreover, the probability of survival after 2 years of follow-up was 67% for subjects without E_2_ and 13% for subjects with low, intermediate and high E_2_ levels. The present article provides evidence that E_2_ plays a role on the survival of patients with MM following therapy. In *in vitro,* we confirmed the presence of E_2_ in MPM cell lines. Furthermore, the preclinical study conducted *in vivo* in mice mesothelioma xenografts, it showed both a reduction of tumour mass following treatment with exemestane and a reduction in plasma levels of E_2_. In mice mesothelioma xenografts untreated with exemestane, an increase of plasmatic E_2_ levels corresponded to increasing tumour mass. It is possible to speculate that E_2_ is necessary for the growth of the tumour, which in turn produces further E_2_. This cycle can be inhibited from exemestane which causes a reduction of E_2_ with consequent reduction of the tumour mass and then E_2_.

In summary, to the best of our knowledge we have identified for the first time differential citoplasmatic E_2_ expression in normal and malignant mesothelium tissues and survival differences by E_2_ status. We found a trend toward a negative correlation between E_2_ levels and the median post-diagnosis survival time. E_2_ was detected in MPM cell lines and in mice mesothelioma xenografts, exemestane therapy induced a reduction of tumour masses and plasmatic E_2_ levels. GPR30 was identified in normal mesothelium while in mesothelioma tissues it was not detected. The presence of GPR30 glycosylated form in MPM cell lines correlated with sensitivity of cell lines to exemestane treatment. Although we were unable to identify GPR30 in tissues at the cellular level, it seems clear that GPR30 and E_2_ in MPM cell lines were involved in mesothelioma proliferation. All together, suggest that MPM cells produce E_2_ what interacts with glycosylated forms of GPR30 and this does grow tumour. The E_2_ reduction inhibits this pathway and leads to cell death and tumour shrinkage. These findings are encouraging and possibly support further investigation of exemestane in the clinical MPM context as well as highlighting the opportunity to test new compounds, which are active with the same mechanism of action, in the experimental MPM model.

## MATERIALS AND METHODS

### Patients and tissue samples

Fifty-seven confirmed cases of MPM and three control subjects were identified from the archival pathology files of the Pathology Unit of the Regional Hospital of Mestre-Venice, Italy. All diagnoses of MPM were based on World Health Organization criteria and confirmed in all instances by clinical, morphological, and immunohistochemistry data, according the recent guidelines for diagnosis of malignant mesothelioma. The tissue samples were from videothoracoscopic biopsy or from surgical specimens. The tissue samples were fixed in neutral formalin and embedded in paraffin. Permission for tissue to be used for research purposes was obtained according to local ethical procedures and following informed patient consent.

Clinical data relating to each of the subjects were obtained with consent from primary patient records and coded before analysis by researchers.

### Immunohistochemistry

Immunohistochemistry parameters for all the specific-antibodies were initially optimized using a breast carcinoma and placenta. Immunohistochemistry analysis for each antigen was performed using a Bond III Automated IHC Stainer (Leica Microsystems, Wetzlar, Germany) on serial 4-_m depth tissue sections from each of the embedded specimens. Slides were treated for 20 minutes with Leica BondMax Epitope Retrieval Solution for detection of ER alpha and beta, 17-beta estradiol and GPR30 to achieve post-sectioning antigen retrieval. Specific primary antibodies were applied as indicated: ER beta (ERBeta-14C8, mouse monoclonal antibody, 1:50; Gene Tex; ER alpha (Clone F-10, mouse monoclonal antibody, 1:50; Santa Cruz Biotechnology); 17-beta-estradiol (rabbit polyoclonal antibody, ready to use; Biogenex) ER (Clone ER88, mouse monoclonal antibody, 1:200; BioGenex Laboratories, San Ramon, CA), and GPR30 (rabbit polyoclonal antibody, 1:100; Lifespan Biosciences K-19, rabbit polyoclonal antibody, 1:100; Santa Cruz Biotechnology). Primary antibodies were revealed using the Leica Bond Polymer Refine detection kit, and the signal was enhanced using the Leica BondMax DAB enhancer kit. Slides were counterstained with hematoxylin before mounting and microscopic visualization

### Scoring system

Semiquantitative determination of E_2,_ ER, ERα, ERβ and GPR30 was performed. The proportion of positive stained cells was rated as 0 (negative) 1+ (up to 30%), 2+ up to 60% and 3+ more than 60% of positive cells. Slides were independently evaluated and scored in a blind fashion by two independent observers. Any discrepancies in scoring between the observers were resolved by review of the slides under a double-headed microscope, and a consensus score was allocated.

### Cell lines and reagents

The human pleural MM cell lines MSTO-211H (MSTO) and NCIH-2452 (NCI) and mesothelium cell line Met-5A were purchased from the American Type Culture Collection (ATCC) (Rockville, Md). Ist-Mes1, Ist-Mes2, and MPP89 were purchased from Genova Institute Culture Collection. To ensure that the lines are uncontaminated and correctly identified, cell lines were periodically tested for mycoplasma contamination by MycoFluor™ Mycoplasma Detection Kit (Thermo Fischer) and cultured as described previously [32]. Cell morphology was monitored routinely and compared to cell morphology images, growth curve analysis was evaluated periodically. Authentication testing by DNA-fingerprinting was not performed. Before hormonal analysis, all cell lines were gradually conditioned in red phenol free DMEM/F12 supplemented with 10% FBS charcoaled and antibiotics. Exemestane was purchased from Sequoia Research, G15 and E_2_ from Sigma. Estradiol 17β EIA kit (ALPCO Diagnostics, Windham, NH) was used for the quantitative determination of 17-β E_2_ in cells and plasma.

Commercially available antibodies were used for immunodetection of: GPR30 (Lifespan biosciences), Estradiol (Biogenex) and γ-tubulina (Sigma, Saint Louis Missouri, USA), anti-mouse and anti-rabbit secondary antibodies (Santa Cruz Biothechnology).

### Cell treatment

Cells were plated in red phenol free DMEM/F12 supplemented with 10% FBS charcoaled and antibiotics. After 24 h, exemestane or DMSO at the same final concentration of that present in medium with drug were added. The expansion of culture cell proliferation was quantified by manual cell counting at 48 h. Supernatant fluids were harvested and frozen at −80° to quantify E_2_ levels. Experiments were repeated in triplicate and media values were calculated.

### GPR30 inactivation

Cells were plated in red phenol free DMEM/F12 supplemented with 10% FBS charcoaled and antibiotics. After 24 h the cells were treated with G15 (10 nM) or E_2_ (10 nM) or G15 (10 nM) + E_2_ (10 nM). G15 was added 10 minutes before E_2_ (10 nM). The expansion of culture cell proliferation was quantified by manual cell counting at 48 h. Experiments were repeated in triplicate and media values were calculated.

### *In vivo* animal models

Male nude mice (6–8 weeks old; weight 18–25 g) were obtained from Charles River. Mice were housed in the animal facility of the Regina Elena National Cancer Institute for 2 weeks before each experiment; animals had ad libitum water and food. The Ethics Committee of the Cancer Institute approved all the experimental protocols that were carried out in accordance with Italian regulations and with the Guide for the Care and Use of Laboratory Animals. A mouse xenograft model of mesothelioma was created as described previously [10]. MSTO cell suspensions (2.5 × 10^6^) in 0, 2 ml of complete medium were injected subcutaneously into the flank of CD1 nude mice (*n* = 10/treatment group) and growth was measured twice weekly with calipers and calculated by the formula: 4/3 π (large diameter) × (small diameter)^2^. After the establishment of palpable lesions (average diameter > 5 mm), mice were assigned to one of the following treatment groups: 1) Control, 2) Exemestane (8.25 mg/Kg, intraperitoneal (i.p.) 5 days a week. Experimental groups were treated for 60 days. Mice were followed for tumour size and blood sampling from the tail were performed after 29 and 50 days from the beginning of treatment with exemestane.

### E_2_ determination

Estradiol-17β-EIA kit was used for quantitative determination of E_2_ in culture supernatant and mouse plasma. This kit was based on a competitive enzyme immunoassay for quantitative determination of E_2_. Briefly, a fixed amount of E_2_ labelled with horseradish peroxidase (HRP) competes with unlabeled E_2_ present in standards or samples for a limited number of binding sites of a specific antibody. The E_2_-HRP-antibody complex is simultaneously fixed on the wells of the microtiter plate coated with an excess of anti-rabbit-gamma globulins. After incubating for 2 hours at room temperature the microtiter plate is washed to stop the competition reaction. The chromogen solution tetramethylbenzidine (TMB in substrate buffer) is added and incubated for 30 minutes. The reaction is stopped with H_2_SO4 and the absorbance is measured at the appropriate wavelength. The intensity of this coloured product is inversely proportional to the concentration of E_2_ present in the original specimen.

### Western blot analysis

Briefly, 25–50 μg of proteins extracted as described previously [33] from cultured cells were separated by SDS-PAGE and transferred onto nitrocellulose membranes. Membranes were blocked and blotted with relevant antibodies.

### Statistics

Cell culture–based assays were repeated at least 3 times; mean ± SD was calculated. Cell lines were examined separately. Differences in xenograft tumour size *in vivo* were assessed using a 2-tailed Student's *t* test. Significance was set at *P* < 0.05.
